# Hypertension prevalence as a function of different guidelines, India

**DOI:** 10.2471/BLT.19.234500

**Published:** 2019-09-17

**Authors:** Manisha Dubey, Sanjay Rastogi, Ashish Awasthi

**Affiliations:** a World Food Programme, New Delhi, India.; b Indian Institute of Foreign Trade, New Delhi, India.; c Centre for Chronic Conditions and Injuries, Public Health Foundation of India, Plot No. 47, Sector 44, Institutional Area, Gurugram, 122002, India.

## Abstract

**Objective:**

To determine the effect of different hypertension management guidelines and of basing diagnosis on a single reading of blood pressure on the hypertension prevalence in the Indian population.

**Methods:**

We performed a secondary analysis of data acquired as part of the *Fourth national family health survey*, 2015 to 2016, over all districts in India. We calculated the proportion of the population within three different age groups (18 to 34, 35 to 49 and 18 to 49 years of age) with raised blood pressure according to six different guidelines, and how prevalence changed if diagnoses were based on a single blood pressure measurement.

**Findings:**

We observed that the Government of India and the American College of Cardiology/American Heart Association guidelines consistently yielded the lowest and highest prevalence of raised blood pressure; in the combined age group, we calculated the proportion of the population categorized as having raised blood pressure as 7.5% (95% confidence interval (CI): 7.4 to 7.7) and 40.1% (95% CI: 39.7 to 40.7), respectively. When basing diagnosis on a single reading of blood pressure only, a total of 56 million individuals would be erroneously categorized as hypertensive following the Government of India guidelines. We also showed that prevalence of hypertension in India varies with guidelines adhered to; in the combined age group, the national hypertension prevalence was three times higher when following the American College of Cardiology/American Heart Association compared with the Government of India guidelines.

**Conclusion:**

To optimize current clinical practice, health-care providers need to follow universally agreed, evidence-based methods of diagnosing hypertension.

## Résumé

**Objectif:**

Déterminer l'effet de différentes directives de gestion de l'hypertension et de l'établissement d'un diagnostic à partir d'une seule mesure de la tension artérielle sur la prévalence de l'hypertension dans la population indienne.

**Méthodes:**

Nous avons effectué une analyse secondaire des données collectées lors de la quatrième enquête nationale sur la santé des familles, de 2015 à 2016, dans tous les districts d'Inde. Nous avons calculé la part de la population, dans trois tranches d'âge différentes (18-34 ans, 35-49 ans et 18-49 ans), qui présentait une tension artérielle élevée selon six directives différentes, et la manière dont la prévalence changeait si le diagnostic se fondait sur une seule mesure de la tension artérielle.

**Résultats:**

Nous avons observé que les directives du gouvernement indien et celles de l'American College of Cardiology/American Heart Association donnaient systématiquement la prévalence la plus faible et la prévalence la plus élevée d'hypertension artérielle; dans la tranche d'âge combinée, nous avons calculé que la part de la population classée comme souffrant d'hypertension s'élevait respectivement à 7,5% (intervalle de confiance (IC) de 95%: 7,4 à 7,7) et à 40,1% (IC de 95%: 39,7 à 40,7). En fondant le diagnostic uniquement sur une mesure de la tension artérielle, un total de 56 millions de personnes seraient classées à tort comme hypertendues selon les directives du gouvernement indien. Nous avons également montré que la prévalence de l'hypertension en Inde varie en fonction des directives auxquelles on se réfère; dans la tranche d'âge combinée, la prévalence nationale de l'hypertension était trois fois plus élevée si l'on suivait les directives de l'American College of Cardiology/American Heart Association que celles du gouvernement indien.

**Conclusion:**

Pour optimiser la pratique clinique, les prestataires de soins doivent suivre des méthodes de diagnostic de l'hypertension éprouvées et universellement admises.****

## Resumen

**Objetivo:**

Determinar el efecto de las diferentes directrices para el tratamiento de la hipertensión y de basar el diagnóstico en una sola lectura de la presión arterial sobre la prevalencia de la hipertensión en la población india.

**Métodos:**

Se realizó un análisis secundario de los datos adquiridos como parte de la Cuarta Encuesta Nacional de Salud Familiar, 2015-2016, en todos los distritos de la India. Se calculó la proporción de la población dentro de tres grupos de edad diferentes (de 18 a 34 años, de 35 a 49 años y de 18 a 49 años) con presión arterial elevada de acuerdo con seis directrices diferentes, y cómo la prevalencia cambió si el diagnóstico se basó en una sola medición de la presión arterial.

**Resultados:**

Se observó que las directrices del Gobierno de la India y del Colegio Americano de Cardiología/Asociación Americana del Corazón arrojaron sistemáticamente la prevalencia más baja y más alta de presión arterial elevada; en el grupo de edad combinado, se calculó que la proporción de la población categorizada como con presión arterial elevada era de 7,5 % (intervalo de confianza [IC] del 95 %: 7,4 a 7,7) y 40,1 % (IC del 95 %: 39,7 a 40,7), respectivamente. Al basar el diagnóstico en una sola lectura de la presión arterial, un total de 56 millones de personas serían erróneamente clasificadas como hipertensivas según las directrices del Gobierno de la India. También se demostró que la prevalencia de la hipertensión en la India varía según las directrices a las que se adhieren; en el grupo de edad combinado, la prevalencia nacional de la hipertensión era tres veces mayor cuando se seguía las directrices del Colegio Americano de Cardiología/Asociación Americana del Corazón en comparación con las del Gobierno de la India.

**Conclusión:**

Para optimizar la práctica clínica actual, los profesionales de la atención de la salud deben seguir métodos universalmente acordados y basados en la evidencia para diagnosticar la hipertensión.

## ملخص

**الغرض:**

تحديد تأثير المبادئ التوجيهية المختلفة لإدارة ارتفاع ضغط الدم، وكذلك التشخيص المعتمد على قراءة واحدة لضغط الدم، على انتشار ضغط الدم المرتفع بين السكان في الهند.

**الطريقة:**

قم بإجراء تحليل ثانوي للبيانات التي تم الحصول عليها كجزء من المسح الوطني الرابع لصحة الأسرة، خلال 2015 إلى 2016، في جميع المناطق في الهند. قمنا بحساب نسبة السكان في ثلاث فئات عمرية مختلفة (18 إلى 34 سنة، و35 إلى 49 سنة، و18 إلى 49 سنة)، والتي تعاني من ارتفاع ضغط الدم، وذلك وفقا لستة مبادئ توجيهية مختلفة، فضلاً عن مدى التغير في الانتشار إذا كان التشخيص معتمداً على قياس ضغط الدم مرة واحدة.

**النتائج:**

لاحظنا أن المبادئ التوجيهية لكل من الحكومة الهندية، والجمعية الأمريكية لأمراض القلب/جمعية القلب الأمريكية، قد أدت بشكل متسق إلى أدنى وأعلى معدل لانتشار ضغط الدم المرتفع؛ في الفئة العمرية المجمعة، قمنا بحساب نسبة السكان الذين تم تصنيفهم بأنهم يعانون من ارتفاع في ضغط الدم بنسبة 7.5% (فاصل الثقة 95%: 7.4 إلى 7.7) و40.1% (فاصل ثقة 95%: 39.7 إلى 40.7)، على التوالي. عند اعتماد التشخيص على قراءة واحدة فقط لضغط الدم، فسوف يتم تصنيف إجمالي 56 مليون نسمة بشكل خاطئ بأنهم مصابين بارتفاع ضغط الدم وفقاً للمبادئ التوجيهية للحكومة الهندية. كما أوضحنا أن انتشار ضغط الدم المرتفع في الهند يختلف وفقاً للمبادئ التوجيهية المتبعة؛ في الفئة العمرية المجمعة، فإن ارتفاع ضغط الدم على المستوى الوطني كان أعلى ثلاث مرات عنه عند اتباع الجمعية الأمريكية لأمراض القلب/جمعية القلب الأمريكية، مقارنةً بالمبادئ التوجيهية للحكومة الهندية.

**الاستنتاج:**

لتحسين الممارسة السريرية الحالية، يحتاج مقدمو الرعاية الصحية إلى اتباع الطرق المتفق عليها عالمياً، والمعتمدة على الأدلة، لتشخيص الإصابة بارتفاع ضغط الدم. ****

## 摘要

**目的:**

旨在确定不同高血压管理指南和基于单一血压读数的诊断对印度人群高血压患病率的影响。

**方法:**

我们对印度所有地区 2015-2016 年第四次全国家庭健康调查的数据进行了次级分析。根据六个不同指南，我们计算了在三个不同年龄组（18 至 34 岁、35 至 49 岁和 18 至 49 岁）中血压升高人群的比例，以及如果诊断是基于单一血压测量的话，患病率是如何变化的。

**结果:**

我们发现，印度政府和美国心脏病学院/美国心脏协会指南得出的血压升高的最低患病率和最高患病率数据一致；在综合年龄组中，我们计算出血压升高人群的比例分别为 7.5% （95% 置信区间：7.4-7.7）和 40.1% （95% 置信区间：39.7-40.7）。根据印度政府指南，当仅根据单次血压读数进行诊断时，共计会有 5600 万人会被错误归为高血压人群。我们还发现，印度的高血压患病率因遵循不同指南而变化；在综合年龄组中，当遵循美国心脏病学院/美国心脏协会指南时，全国范围内的高血压患病率要比遵循印度政府的准则时的患病率高出三倍。

**结论:**

为了优化目前的临床实践，医护人员应遵循世界公认、基于证据的方法进行高血压的诊断。****

## Резюме

**Цель:**

Определение влияния различных руководящих принципов по ведению больных с артериальной гипертонией и постановки диагноза по однократному измерению артериального давления на показатели распространенности гипертонии среди населения Индии.

**Методы:**

Авторы провели дополнительный анализ данных, полученных в рамках четвертого национального опроса о здоровье семьи, который проводился в 2015–2016 годах во всех регионах Индии. Была рассчитана доля лиц с повышенным артериальным давлением в популяции трех возрастных групп (от 18 до 34 лет, от 35 до 49 лет и от 18 до 49 лет) в соответствии с шестью различными руководящими принципами, а также продемонстрировано изменение показателя распространенности гипертонии, если диагностика опиралась на однократное измерение артериального давления.

**Результаты:**

Авторы обнаружили, что руководящие принципы Правительства Индии и Американской коллегии кардиологов или Американской ассоциации сердца в результате неизменно дают минимальный и максимальный показатели распространенности повышенного артериального давления; в комбинированной возрастной группе, по расчетам авторов, доля популяции с повышенным артериальным давлением составила соответственно 7,5% (95%-й ДИ: 7,4–7,7) и 40,1% (95%-й ДИ: 39,7–40,7). В случае постановки диагноза на основании однократного измерения артериального давления 56 миллионам людей был бы ошибочно поставлен диагноз гипертонии на основании руководящих принципов Правительства Индии. Авторы также доказали, что уровень распространенности гипертонии в Индии зависит от того, каким руководящим принципам следуют врачи: в комбинированной возрастной группе национальный уровень распространенности гипертонии был в три раза выше при использовании руководящих принципов Американской коллегии кардиологов или Американской ассоциации сердца, чем при использовании руководящих принципов Правительства Индии.

**Вывод:**

Для оптимизации текущей клинической практики работники здравоохранения должны следовать всесторонне согласованным и научно обоснованным методам диагностики гипертонии.

## Introduction

With developments in technology and the expansion of treatment options and modalities, the field of clinical care guidelines is constantly evolving. Although clinical care guidelines are only recommendations, the decision to follow a specific set of guidelines by a health-care provider should be based on the local context of need, availability and affordability, especially in low- and middle-income countries.^1^ The availability of different guidelines, with inconsistencies in recommendations of when medical treatment should be initiated, can cause friction between health-care provider and patient.^2^^,^^3^

Blood pressure measurement is one of the most common non-invasive clinical practice tools used to assess the cardiovascular status of an individual and predict the likelihood of future cardiovascular events. An individual’s blood pressure can change quickly and regularly, and is influenced by respiration, temperature, bladder distension, pain levels, emotion, diet, time since last exercise and whether alcohol has recently been consumed.^4^ A simple method of obtaining an accurate blood pressure measurement is to take repeated readings over multiple visits. Compared with diagnoses of hypertension based on a single measurement of blood pressure, studies have demonstrated as much as 12% reduction in the prevalence of hypertension if repeated readings over multiple visits are considered.^5^^,^^6^ By considering multiple readings, up to 35% of patients were reclassified within a lower category of blood pressure.^5^^,^^6^ However, even after obtaining a more accurate blood pressure measurement, clinical care guidelines differ with respect to the precise blood pressure at which a patient is diagnosed as hypertensive and begins treatment.^7^^–^^12^ This lack of uniformity between the various available guidelines diminishes the value of measuring blood pressure.

Over the past 25 years, the availability of health-care services have increased in India, and the country has adopted a universal health coverage programme.^13^ Although the availability of health-care services has risen, the quality of treatment received from different health-care providers is not consistent.^1^^,^^14^^–^^16^ The causes of this inconsistency in quality across India include variations in clinical practice, poor diagnostic facilities, a lack of expertise, unnecessary use of medicines (e.g. antibiotics, analgesics and steroids) and substandard treatment.^1^ India is currently experiencing an increase in the prevalence of noncommunicable diseases, such as hypertension and diabetes, and the accompanying premature mortality.^17^^–^^21^ Inconsistent guidelines introduce uncertainty in the accuracy of hypertension diagnoses and increase the likelihood of poor health outcomes.^22^ Poor health-care literacy, high self-medication rate, poor blood pressure control and inconsistent hypertension management guidelines intensify the problem in India.^23^

Here we have analysed the impact of inconsistent practices on the calculated prevalence of hypertension. We have focused on the particular blood pressure measurements at which hypertension is diagnosed and whether a single reading or the recommended number of readings was taken, for six different hypertension guidelines.

## Methods

### Data source

We used data from the most recent large-scale health survey, the Fourth National Family Health Survey,^24^ conducted over 2015 to 2016 in India. The Fourth National Family Health Survey was conducted over all 640 districts of India (according to Census of India 2011 listing),^25^ and included men aged 15 to 54 years and women aged 15 to 49 years. For consistency in our study, we included participants aged 18 to 49 years. Households within each district were selected to participate in the survey by two-stage cluster random sampling, stratified by rural versus urban areas. Primary sampling units, selected using probability proportional to population size, were defined as villages in rural areas and census enumeration blocks in urban areas. After sitting calmly for 5 minutes, the blood pressure of participants was measured three times, with at least 5 minutes between each measurement, in the left upper arm using the Omron HEM-8712 monitor. All blood pressure measurements were recorded in millimetres of mercury (mm Hg).

### Hypertension guidelines

The six different guidelines that we used in this study for the calculation of hypertension prevalence are published by the European Society of Cardiology,^7^ the Government of India,^8^ the American College of Cardiology/American Heart Association,^9^ the National Institute for Health and Clinical Excellence/British and Irish Hypertension Society,^10^ the Eighth Joint National Committee^11^ and the International Society of Hypertension.^12^ The latter two guidelines are identical in terms of diagnosis of raised blood pressure. All six guidelines are used in India for the diagnosis and treatment of hypertension; although the exact proportion of health-care providers across India that adhere to any particular guideline is not known, the proportion of health-care providers adhering to specific guidelines was investigated within a single private hospital^26^ and among attendees of a cardiology conference.^27^

The number of readings taken, the number of visits required by the patient and the blood pressure at which hypertension is diagnosed (and pharmacological antihypertensive treatment initiated) for each of these six guidelines are listed in Table 1. Although all the listed guidelines recommend the measurement of blood pressure from at least two or three readings, it is general practice in India to take only a single blood pressure reading.^28^ A patient is defined as having raised blood pressure if their blood pressure is categorized as Stage 1 or Grade 1 hypertension or higher according to the different guidelines in Table 1. A patient is defined as hypertensive if they have raised blood pressure, have confirmed at the time of the survey that they were taking prescribed medicine to control blood pressure, or if they had previously received at least two diagnoses of raised blood pressure or hypertension.

**Table 1:** Guidelines for definition of raised blood pressure used in comparison study, India

No. of readings by guideline	Reading considered	Hypertension diagnosis	Blood pressure category^a^		Blood pressure (mm Hg)	
					Systolic	Diastolic
**European Society of Cardiology/European Society of Hypertension**^7^						
At least three (if different by ≥ 10 mm Hg, at least four)	Average of last two readings	Other than Grade 3, two visits required	Optimal^b^		< 120	< 80
			Normal		120 to 129	80 to 84
			High normal		130 to 139	85 to 89
			**Grade 1 hypertension**		**140 to 159**	**90 to 99**
			Grade 2 hypertension		160 to 179	100 to 109
			Grade 3 hypertension		≥ 180	≥ 110
			Isolated systolic hypertension		≥ 140	< 90
**Government of India**^8^						
At least two (if different by ≥ 5 mm Hg, at least three)	Lowest	Other than Grade 3, two visits required	Optimal^b^		< 120	< 80
			Normal		120 to 129	80 to 84
			High normal		130 to 139	85 to 89
			**Grade 1 hypertension**		**140 to 159**	**90 to 99**
			Grade 2 hypertension		160 to 179	100 to 109
			Grade 3 hypertension		≥ 180	≥ 110
			Isolated systolic hypertension^b^		≥ 140	< 90
			Hypertensive urgency		> 180	> 110
			Hypertensive emergency		> 180	> 110 to 120
**American College of Cardiology/American Heart Association**^9^						
At least two	Average	Two visits or more	Normal^b^		< 120	< 80
			Elevated^b^		120 to 129	< 80
			**Stage 1 hypertension^c^**		**130 to 139**	**80 to 89**
			Stage 2 hypertension		≥ 140	≥ 90
**National Institute for Health and Clinical Excellence/British and Irish Hypertension Society**^10^						
At least two (if readings different, at least three)	Average of last two readings	Two visits or more	Normal^b^		< 135	< 85
			**Stage 1 hypertension**		** ≥ 135**	** ≥ 85**
			Stage 2 hypertension		≥ 150	≥ 95
			Severe hypertension		≥ 180	≥ 110
**Eighth Joint National Committee**^11^						
At least two	Average	Two visits or more	Normal^b^		< 120	< 80
			Prehypertension		120 to 139	80 to 89
			**Stage 1 hypertension**		**140 to 159**	**90 to 99**
			Stage 2 hypertension		≥ 160	≥ 100
**International Society of Hypertension**^12^						
At least two	Average	Two visits or more	Normal^b^		< 120	< 80
			Prehypertension		120 to 139	80 to 89
			**Stage 1 hypertension**		**140 to 159**	**90 to 99**
			Stage 2 hypertension		≥ 160	≥ 100

Hg: mercury.

^a^ Bold formatting indicates the blood pressure category at which medical treatment is initiated.

^b^ Categories for which both systolic and diastolic blood pressure measurements of less than threshold are required; patients are assigned to other categories if either of their systolic or diastolic blood pressure measurement is within the given limit.

^c^ Medical treatment initiated only if the patient has a Framingham risk score (risk of developing cardiovascular disease over the next 10 years) of ≥ 10%.

### Statistical analysis

From the sample, we excluded participants having at least one missing blood pressure measurement or having unfeasible (i.e. systolic blood pressure < 30 mm Hg or < diastolic blood pressure) readings. We calculated the proportion of individuals within various blood pressure categories for age groups 18 to 34, 35 to 49 and 18 to 49 years of age for all six guidelines. We applied sampling weights and adjusted confidence intervals (CIs) at the primary sampling unit level to obtain nationally representative estimates with precise CIs. 

To calculate prevalence, we defined participants having hypertension if they had stage I/grade I or higher blood pressure, taking prescribed medicine to control blood pressure or being informed at least twice by the health professional that they had raised blood pressure or hypertension. We estimated the total population within each age group from Census of India 2011 age distribution data,^25^ multiplied by World Bank Indian population estimates for the year 2017.^29^

We calculated the number of individuals across India within each category for each guideline by multiplying the proportion within each blood pressure category according to the Fourth National Family Health Survey by the calculated population within each age group. To confirm that the exclusion of participants did not cause significant difference in terms of age, sex and place of residence in the final data set, we performed a sensitivity analysis by comparing prevalence estimates from the clean data set with those from the full data set. We performed all analyses using Stata software, version 15.0 (StataCorp, College Station, United States of America).

### Ethics

The Fourth National Family Health Survey obtained ethical clearance from the Ethics Committee of the International Institute for Population Sciences.^24^ No specific permission was required for our study, as we conducted a secondary analysis of publicly available data.

## Results

We obtained data on 797 161 individuals from the survey. We excluded 45 691 patients with missing data and 1594 participants with unusual blood pressure measurements.^24^ Of the 749 876 eligible participants, 651 605 (86.9%) were women and 98 271 (13.1%) were men, and 529 899 (70.7%) of individuals resided in rural areas. Our study sample comprises 439 414 (58.6%) individuals 18 to 34 years of age and 310 462 (41.4%) individuals 35 to 49 years of age. The sensitivity analysis showed that the exclusion of participants with missing or unfeasible readings did not cause a difference in the final data set.

We observe that the Government of India and the American College of Cardiology/American Heart Association guidelines consistently yield the lowest and highest prevalence of measured raised blood pressure, respectively (Table 2). For the combined age group, in order of increasing prevalence the weighted proportion of the population classified as having raised blood pressure is: 7.5% (95% CI: 7.4 to 7.7; Government of India guidelines); 10.1% (95% CI: 10.0 to 10.2; European Society of Cardiology/European Society of Hypertension guidelines); 13.1% (95% CI: 13.1 to 13.3; Eighth Joint National Committee and International Society of Hypertension guidelines); 19.4% (95% CI: 19.3 to 19.6; National Institute for Health and Clinical Excellence/British and Irish Hypertension Society guidelines); and 40.1% (95% CI: 39.7 to 40.7; American College of Cardiology/American Heart Association guidelines; Table 2). Among the group18 to 34 years of age, the Government of India and the American College of Cardiology/American Heart Association guidelines yielded proportions of the population with raised blood pressure of 3.4% (95% CI: 3.3 to 3.5) and 30.3% (95% CI: 30.2 to 30.5), respectively. Among the older age group, the lowest and highest proportions were calculated as 13.8% (95% CI: 13.7 to 14.0; Government of India guidelines) and 55.3% (95% CI: 55.2 to 55.5; American College of Cardiology/American Heart Association guidelines), respectively. Following the guidelines set by the Government of India, we estimate 48 million Indians have raised blood pressure; if the American College of Cardiology/American Heart Association guidelines are followed, this number is 253 million (Table 3).

**Table 2:** Proportion of population with a blood pressure level according to category, guideline and reading, India, 2015–2016

Blood pressure category by guideline	Weighted % (95% CI)^a^						
	If guidelines followed				If only first reading considered		
	18 to 34 years	35 to 49 years	18 to 49 years		18 to 34 years	35 to 49 years	18 to 49 years
**European Society of Cardiology/European Society of Hypertension**^7^							
Optimal	66.0 (65.7 to 66.2)	41.5 (41.1 to 41.8)	56.4 (56.1 to 56.6)		53.3 (53.0 to 53.6)	31.4 (31.1 to 31.7)	44.7 (44.5 to 45.0)
Normal	20.4 (20.1 to 20.6)	24.7 (24.5 to 25.0)	22.1 (21.9 to 22.3)		23.1 (22.9 to 23.3)	22.8 (22.5 to 23.1)	23.0 (22.8 to 23.2)
High normal	8.4 (8.3 to 8.6)	16.1 (15.9 to 16.4)	11.5 (11.3 to 11.6)		13.4 (13.2 to 13.6)	19.5 (19.3 to 19.8)	15.8 (15.7 to 16.0)
Grade 1 hypertension	3.9 (3.8 to 4.0)	11.3 (11.1 to 11.5)	6.8 (6.7 to 6.9)		7.3 (7.1 to 7.4)	16.2 (16.0 to 16.4)	10.7 (10.6 to 10.9)
Grade 2 hypertension	0.6 (0.6 to 0.7)	3.1 (3.0 to 3.2)	1.6 (1.6 to 1.7)		1.2 (1.2 to 1.3)	4.8 (4.6 to 4.9)	2.6 (2.6 to 2.7)
Grade 3 hypertension	0.1 (0.1 to 0.2)	1.2 (1.1 to 1.2)	0.5 (0.5 to 0.6)		0.4 (0.4 to 0.5)	2.0 (1.9 to 2.1)	1.0 (1.0 to 1.1)
Isolated systolic hypertension	0.6 (0.5 to 0.6)	2.0 (2.0 to 2.1)	1.1 (1.1 to 1.2)		1.3 (1.2 to 1.3)	3.3 (3.2 to 3.4)	2.1 (2.0 to 2.1)
**Government of India**^8^							
Optimal	73.7 (73.4 to 73.9)	49.2 (48.8 to 49.5)	64.1 (63.8 to 64.3)		53.3 (53.0 to 53.6)	31.4 (31.1 to 31.7)	44.7 (44.5 to 45.0)
Normal	17.1 (16.9 to 17.3)	23.9 (23.6 to 24.1)	19.7 (19.5 to 19.9)		23.1 (22.9 to 23.3)	22.8 (22.5 to 23.1)	23.0 (22.8 to 23.2)
High normal	5.8 (5.7 to 5.9)	13.1 (12.9 to 13.3)	8.7 (8.5 to 8.8)		13.4 (13.2 to 13.6)	19.5 (19.3 to 19.8)	15.8 (15.7 to 16.0)
Grade 1 hypertension	2.6 (2.6 to 2.7)	8.8 (8.7 to 9.0)	5.1 (5.0 to 5.2)		7.3 (7.1 to 7.4)	16.2 (16.0 to 16.4)	10.7 (10.6 to 10.9)
Grade 2 hypertension	0.4 (0.4 to 0.4)	2.4 (2.3 to 2.5)	1.2 (1.1 to 1.2)		1.2 (1.2 to 1.3)	4.8 (4.6 to 4.9)	2.6 (2.6 to 2.7)
Grade 3 hypertension	0.1 (0.1 to 0.1)	0.8 (0.8 to 0.9)	0.4 (0.4 to 0.4)		0.4 (0.4 to 0.5)	2.0 (1.9 to 2.1)	1.0 (1.0 to 1.1)
Isolated systolic hypertension	0.3 (0.3 to 0.3)	1.8 (1.7 to 1.9)	0.9 (0.9 to 0.9)		1.3 (1.2 to 1.3)	3.3 (3.2 to 3.4)	2.1 (2.0 to 2.1)
**American College of Cardiology/American Heart Association**^9^							
Normal	63.3 (63.0 to 63.6)	38.7 (38.3 to 39.0)	53.6 (53.4 to 53.9)		53.3 (53.0 to 53.6)	31.4 (31.1 to 31.7)	44.7 (44.5 to 45.0)
Elevated	6.4 (6.3 to 6.5)	6.1 (5.9 to 6.2)	6.3 (6.2 to 6.4)		7.2 (7.0 to 7.3)	5.9 (5.8 to 6.1)	6.7 (6.6 to 6.8)
Stage 1 hypertension	24.5 (24.3 to 24.8)	36.2 (35.9 to 36.5)	29.1 (28.9 to 29.3)		29.4 (29.1 to 29.6)	36.4 (36.1 to 36.7)	32.1 (31.9 to 32.3)
Stage 2 hypertension	5.8 (5.7 to 5.9)	19.1 (18.8 to 19.3)	11.0 (10.9 to 11.1)		10.2 (10.0 to 10.3)	26.3 (26.0 to 26.5)	16.5 (16.3 to 16.6)
Stage 1 with Framingham risk score of ≥ 10%^b^	8.4 (8.3 to 8.6)	54.1 (53.7 to 54.4)	30.4 (30.1 to 30.6)		10.9 (10.7 to 11.1)	61.2 (60.8 to 61.5)	36.3 (36.0 to 36.6)
**National Institute for Health and Clinical Excellence/British and Irish Hypertension Society**^10^							
Normal	87.9 (87.7 to 88.1)	69.2 (68.9 to 69.5)	80.6 (80.4 to 80.8)		78.6 (78.3 to 78.8)	57.2 (56.9 to 57.5)	70.2 (70.0 to 70.4)
Stage 1 hypertension	10.1 (9.9 to 10.3)	22.0 (21.7 to 22.2)	14.7 (14.6 to 14.9)		17.1 (16.9 to 17.3)	28.7 (28.5 to 29.0)	21.6 (21.5 to 21.8)
Stage 2 hypertension	1.9 (1.8 to 1.9)	7.7 (7.5 to 7.8)	4.1 (4.1 to 4.2)		3.9 (3.8 to 4.0)	12.0 (11.8 to 12.2)	7.1 (7.0 to 7.2)
Severe hypertension	0.2 (0.1 to 0.2)	1.2 (1.1 to 1.3)	0.6 (0.5 to 0.6)		0.5 (0.4 to 0.5)	2.0 (2.0 to 2.1)	1.1 (1.0 to 1.1)
**Eighth Joint National Committee**^11^							
Normal	59.5 (59.2 to 59.8)	35.8 (35.5 to 36.2)	50.3 (50.0 to 50.5)		53.5 (53.2 to 53.8)	31.5 (31.2 to 31.8)	44.9 (44.7 to 45.2)
Prehypertension	33.1 (32.9 to 33.4)	42.1 (41.8 to 42.5)	36.7 (36.4 to 36.9)		36.3 (36.0 to 36.5)	42.1 (41.8 to 42.4)	38.6 (38.3 to 38.8)
Stage 1 hypertension	6.2 (6.1 to 6.3)	16.4 (16.2 to 16.6)	10.2 (10.1 to 10.3)		8.4 (8.3 to 8.5)	19.3 (19.0 to 19.5)	12.6 (12.5 to 12.8)
Stage 2 hypertension	1.1 (1.1 to 1.2)	5.6 (5.5 to 5.8)	2.9 (2.8 to 2.9)		1.8 (1.7 to 1.9)	7.1 (6.9 to 7.3)	3.9 (3.8 to 4.0)
**International Society of Hypertension**^12^							
Normal	59.5 (59.2 to 59.8)	35.8 (35.5 to 36.2)	50.3 (50.0 to 50.5)		53.5 (53.2 to 53.8)	31.5 (31.2 to 31.8)	44.9 (44.7 to 45.2)
Prehypertension	33.1 (32.9 to 33.4)	42.1 (41.8 to 42.5)	36.7 (36.4 to 36.9)		36.3 (36.0 to 36.5)	42.1 (41.8 to 42.4)	38.6 (38.3 to 38.8)
Stage 1 hypertension	6.2 (6.1 to 6.3)	16.4 (16.2 to 16.6)	10.2 (10.1 to 10.3)		8.4 (8.3 to 8.5)	19.3 (19.0 to 19.5)	12.6 (12.5 to 12.8)
Stage 2 hypertension	1.1 (1.1 to 1.2)	5.6 (5.5 to 5.8)	2.9 (2.8 to 2.9)		1.8 (1.7 to 1.9)	7.1 (6.9 to 7.3)	3.9 (3.8 to 4.0)

CI: confidence interval; Hg mercury.

^a^ Some columns do not add up to 100% because of rounding of individual percentages to a single decimal point.

^b^ Framingham risk score (risk of developing cardio vascular disease within 10 years) of ≥ 10% as well as systolic blood pressure ≥ 130 mm Hg or diastolic blood pressure ≥ 80 mm Hg.

**Table 3:** Number of people categorized by blood pressure level, guidelines and readings, India, 2015–2016

Blood pressure category by guideline	Population in millions (95% CI)								Difference in millions		
	If guidelines followed				If only first reading considered				18 to 34 years	35 to 49 years	18 to 49 years^a^
	18 to 34 years	35 to 49 years	18 to 49 years^a^		18 to 34 years	35 to 49 years	18 to 49 years^a^				
**European Society of Cardiology/European Society of Hypertension**^7^											
Optimal	256 (254 to 257)	101 (100 to 102)	356 (354 to 357)		207 (205 to 208)	76 (76 to 77)	282 (280 to 284)		−49	−25	−73
Normal	79 (78 to 80)	60 (60 to 61)	139 (138 to 140)		89 (89 to 90)	55 (55 to 56)	145 (144 to 146)		11	−5	6
High normal	33 (32 to 33)	39 (39 to 40)	72 (71 to 73)		52 (51 to 53)	48 (47 to 48)	100 (99 to 101)		19	8	27
Grade 1 hypertension	15 (15 to 16)	27 (27 to 28)	43 (42 to 44)		28 (28 to 29)	39 (39 to 40)	68 (67 to 69)		13	12	25
Grade 2 hypertension	2 (2 to 3)	8 (7 to 8)	10 (10 to 10)		5 (5 to 5)	12 (11 to 12)	16 (16 to 17)		2	4	6
Grade 3 hypertension	1 (1 to 1)	3 (3 to 3)	3 (3 to 4)		2 (1 to 2)	5 (5 to 5)	6 (6 to 7)		1	2	3
Isolated systolic hypertension	2 (2 to 2)	5 (5 to 5)	7 (7 to 7)		5 (5 to 5)	8 (8 to 8)	13 (13 to 13)		3	3	6
**Government of India**^8^											
Optimal	285 (284 to 286)	120 (119 to 120)	404 (402 to 406)		207 (205 to 208)	76 (76 to 77)	282 (280 to 284)		−79	−43	−122
Normal	66 (65 to 67)	58 (57 to 59)	124 (123 to 126)		89 (89 to 90)	55 (55 to 56)	145 (144 to 146)		23	−3	21
High normal	23 (22 to 23)	32 (31 to 32)	55 (54 to 55)		52 (51 to 53)	48 (47 to 48)	100 (99 to 101)		29	16	45
Grade 1 hypertension	10 (10 to 11)	21 (21 to 22)	32 (31 to 33)		28 (28 to 29)	39 (39 to 40)	68 (67 to 69)		18	18	36
Grade 2 hypertension	2 (1 to 2)	6 (6 to 6)	7 (7 to 8)		5 (5 to 5)	12 (11 to 12)	16 (16 to 17)		3	6	9
Grade 3 hypertension	0 (0 to 0)	2 (2 to 2)	2 (2 to 3)		2 (1 to 2)	5 (5 to 5)	6 (6 to 7)		1	3	4
Isolated systolic hypertension	1 (1 to 1)	4 (4 to 5)	6 (5 to 6)		5 (5 to 5)	8 (8 to 8)	13 (13 to 13)		4	4	7
**American College of Cardiology/American Heart Association**^9^											
Normal	245 (244 to 246)	94 (93 to 95)	338 (337 to 340)		207 (205 to 208)	76 (76 to 77)	282 (280 to 284)		−38	−18	−56
Elevated	25 (24 to 25)	15 (14 to 15)	40 (39 to 40)		28 (27 to 28)	14 (14 to 15)	42 (41 to 43)		3	0	3
Stage 1 hypertension	95 (94 to 96)	88 (87 to 89)	183 (182 to 185)		114 (113 to 115)	89 (88 to 89)	202 (201 to 204)		19	1	19
Stage 2 hypertension	22 (22 to 23)	46 (46 to 47)	69 (68 to 70)		39 (39 to 40)	64 (63 to 65)	104 (103 to 105)		17	17	34
Stage 1 with Framingham risk score of ≥ 10%^b^	33 (32 to 33)	132 (131 to 132)	191 (190 to 193)		42 (41 to 43)	149 (148 to 150)	229 (227 to 231)		9	17	37
**National Institute for Health and Clinical Excellence/British and Irish Hypertension Society**^10^											
Normal	340 (340 to 341)	168 (168 to 169)	508 (507 to 509)		304 (303 to 305)	139 (138 to 140)	443 (441 to 444)		−36	−29	−65
Stage 1 hypertension	39 (38 to 40)	53 (53 to 54)	93 (92 to 94)		66 (65 to 67)	70 (69 to 71)	136 (135 to 138)		27	17	44
Stage 2 hypertension	7 (7 to 8)	19 (18 to 19)	26 (26 to 27)		15 (15 to 15)	29 (29 to 30)	44 (44 to 45)		8	11	18
Severe hypertension	1 (1 to 1)	3 (3 to 3)	4 (3 to 4)		2 (2 to 2)	5 (5 to 5)	7 (7 to 7)		1	2	3
**Eighth Joint National Committee**^11^											
Normal	231 (229 to 232)	87 (86 to 88)	317 (315 to 319)		207 (206 to 209)	77 (76 to 77)	283 (282 to 285)		−23	−11	−34
Prehypertension	128 (127 to 129)	102 (102 to 103)	231 (230 to 233)		141 (139 to 142)	102 (102 to 103)	243 (242 to 245)		12	0	12
Stage 1 hypertension	24 (24 to 25)	40 (39 to 40)	64 (64 to 65)		33 (32 to 33)	47 (46 to 47)	80 (79 to 81)		8	7	15
Stage 2 hypertension	4 (4 to 5)	14 (13 to 14)	18 (18 to 19)		7 (7 to 7)	17 (17 to 18)	24 (24 to 25)		3	4	6
**International Society of Hypertension**^12^											
Normal	231 (229 to 232)	87 (86 to 88)	317 (315 to 319)		207 (206 to 209)	77 (76 to 77)	283 (282 to 285)		−23	−11	−34
Prehypertension	128 (127 to 129)	102 (102 to 103)	231 (230 to 233)		141 (139 to 142)	102 (102 to 103)	243 (242 to 245)		12	0	12
Stage 1 hypertension	24 (24 to 25)	40 (39 to 40)	64 (64 to 65)		33 (32 to 33)	47 (46 to 47)	80 (79 to 81)		8	7	15
Stage 2 hypertension	4 (4 to 5)	14 (13 to 14)	18 (18 to 19)		7 (7 to 7)	17 (17 to 18)	24 (24 to 25)		3	4	6

CI: confidence interval; Hg mercury.

^a^ The population in some combined age groups do not always equal the sum of the population in the two subgroups because of rounding to a single decimal point.

^b^ Framingham risk score (risk of developing cardio vascular disease within 10 years) of ≥ 10% as well as systolic blood pressure ≥ 130 mm Hg or diastolic blood pressure ≥ 80 mm Hg.

We also observe an increase in the weighted proportion of the population classified as having raised blood pressure when only a single blood pressure reading (i.e. the first reading taken) is considered, compared with measuring blood pressure from several readings as recommended by the guidelines (Table 2). If the proportion is based on first reading only, the guidelines published by the European Society of Cardiology/European Society for Hypertension and by the Government of India yield the same results. The increase in the proportion is higher in the younger compared with the older age group for all guidelines. Specifically, when we consider only the first reading for blood pressure categorization, the proportion of the population in the combined age group with raised blood pressure according to the Government of India guidelines increases by 8.9 percentage-points to 16.5% (95% CI: 16.5 to 16.7). According to the American College of Cardiology/American Heart Association guidelines, the proportion increases by 8.5 percentage-points to 48.6% (95% CI: 48.5 to 48.8) when only the first blood pressure reading is considered. 

By neglecting to follow any guidelines precisely and by basing diagnosis on a single reading of blood pressure only, a total of 56 million would be erroneously categorized as hypertensive instead of normotensive following the American College of Cardiology/American Heart Association guidelines (Table 3). The largest increase in patients misdiagnosed with raised blood pressure from a single reading (65 million; Table 3) is observed for the National Institute for Health and Clinical Excellence/British and Irish Hypertension Society guidelines.

Table 4 shows the national hypertension prevalence according to various guidelines. For the combined age group, following the American College of Cardiology/American Heart Association guidelines yields a hypertension prevalence (44.7%, 95% CI: 44.4 to 45.0) three times higher than that calculated according to the Government of India guidelines (15.8%, 95% CI: 15.5 to 16.0). 

**Table 4:** National hypertension prevalence by age group and guidelines, India, 2015–2016

Guideline	Weighted % (95% CI)								Population in millions (95% CI)						
	If guidelines followed				If only first reading considered				If guidelines followed				If only first reading considered		
	18 to 34 years	35 to 49 years	18 to 49 years		18 to 34 years	35 to 49 years	18 to 49 years		18 to 34 years	35 to 49 years	18 to 49 years^a^		18 to 34 years	35 to 49 years	18 to 49 years^a^
European Society of Cardiology/ European Society of Hypertension^7^	12.2 (11.9 to 12.5)	26.8 (26.5 to 27.2)	17.9 (17.7 to 18.2)		16.7 (16.4 to 16.9)	34.2 (33.8 to 34.5)	23.5 (23.2 to 23.8)		47 (46 to 48)	65 (64 to 66)	113 (111 to 115)		65 (63 to 66)	83 (82 to 84)	148 (146 to 150)
Government of India^8^	10.6 (10.3 to 10.9)	23.8 (23.5 to 24.2)	15.8 (15.5 to 16.0)		16.7 (16.4 to 16.9)	34.2 (33.8 to 34.5)	23.5 (23.2 to 23.8)		41 (40 to 42)	58 (57 to 59)	99 (98 to 101)		65 (63 to 66)	83 (82 to 84)	148 (146 to 150)
American College of Cardiology/ American Heart Association^9^	35.2 (34.9 to 35.5)	59.6 (59.2 to 59.9)	44.7 (44.4 to 45.0)		43.7 (43.4 to 44.0)	66.2 (65.9 to 66.5)	52.5 (52.2 to 52.8)		136 (135 to 138)	145 (144 to 146)	282 (280 to 284)		169 (168 to 171)	161 (160 to 162)	331 (329 to 333)
National Institute for Health and Clinical Excellence/ British and Irish Hypertension Society^10^	18.4 (18.1 to 18.7)	38.0 (37.6 to 38.3)	26.0 (25.8 to 26.3)		27.0 (26.7 to 27.3)	48.5 (48.2 to 48.9)	35.4 (35.2 to 35.7)		71 (70 to 72)	92 (91 to 93)	164 (162 to 166)		105 (104 to 106)	118 (117 to 119)	223 (222 to 225)
Eighth Joint National Committee^11^	14.1 (13.8 to 14.4)	30.1 (30.0 to 30.9)	20.5 (20.2 to 20.8)		16.7 (16.5 to 17.0)	34.3 (33.9 to 34.7)	23.6 (23.3 to 23.9)		55 (53 to 56)	73 (73 to 75)	129 (128 to 131)		65 (64 to 66)	83 (82 to 84)	149 (147 to 151)
International Society of Hypertension^12^	14.1 (13.8 to 14.4)	30.1 (30.0 to 30.9)	20.5 (20.2 to 20.8)		16.7 (16.5 to 17.0)	34.3 (33.9 to 34.7)	23.6 (23.3 to 23.9)		55 (53 to 56)	73 (73 to 75)	129 (128 to 131)		65 (64 to 66)	83 (82 to 84)	149 (147 to 151)

CI: confidence interval.

^a^ The population in some combined age groups do not always equal the sum of the population in the two subgroups because of rounding to a single decimal point.

## Discussion

This study compares the difference in hypertension prevalence when using six hypertension management guidelines in India. Our findings, that prevalence of hypertension varies according to guidelines followed and according to the number of blood pressure readings taken, are in concordance with other studies.^6^^,^^30^^,^^31^

Another recent study^30^ compared hypertension prevalence in India according to the Seventh Joint National Committee and the American College of Cardiology/American Heart Association guidelines. Their observation is in concordance with ours, that is, that hypertension prevalence more than doubles when calculated according to the American College of Cardiology/American Heart Association guidelines compared with Eight Joint National Committee guidelines.^30^ The other study investigated hypertension prevalence in the age group 30 to 74 years and obtained a prevalence of 52.3% (95% CI; 51.9 to 52.8) according to the American College of Cardiology/American Heart Association guidelines,^30^ similar to our observation of 44.7%. Our findings also show that 30.4% (95% CI: 30.1 to 30.6) of individuals 18 to 49 years of age with Stage 1 hypertension (according to American College of Cardiology/American Heart Association guidelines) have a 10-year risk of developing cardiovascular disease equal to or more than 10%; this figure was calculated as 40.3% for the age group 30 to 74 years in the recent study.^30^ Our study is more versatile, however, with a comparison of six guidelines for hypertension prevalence using both single and repetitive measurements.

Our study had several limitations. We may have overestimated hypertension prevalence by our definition of hypertension being based on blood pressure measurements taken during one occasion; a clinical diagnosis of hypertension requires raised blood pressure on at least two different occasions.^32^ The lower age of participants in this sample is also largely responsible for the lower hypertension prevalence observed here compared with the nationally representative study among an older sample.^18^ Another limitation of our study is the lack of nationally representative data regarding use of hypertension guidelines by health-care providers in India. Finally, the questions asked in the Fourth National Family Health Survey did not allow us to investigate any connection between the prevalence of hypertension and lifestyle.

Our results show that the current use of several different guidelines in India results in inconsistent prevalence data, which could result in poor health outcomes. We therefore urge global bodies to discuss and propose a universal guideline, similar to the cut-off for body mass index, malnutrition and anaemia. In our opinion, the European Society of Hypertension guidelines are most suited for India; these guidelines have the same definitions of blood pressure categories as the Government of India guidelines, but diagnosis is made from the last two readings (out of three) instead of the lowest reading (out of two or three). This recommendation is supported by two different studies.^30^^,^^33^

To optimize current clinical practice in India, health-care providers need to follow universally agreed, evidence-based methods of diagnosing hypertension. The importance of multiple measurements and its impact on health management must be emphasized to health-care professionals. Once such guidelines have been agreed upon, their display at prominent locations within hospitals could help to improve the health literacy of the general population.
